# Prevalence and molecular characterization of *oqxAB* in clinical *Escherichia coli* isolates from companion animals and humans in Henan Province, China

**DOI:** 10.1186/s13756-018-0310-8

**Published:** 2018-02-02

**Authors:** Baoguang Liu, Hua Wu, Yajun Zhai, Zhipei He, Huarun Sun, Tian Cai, Dandan He, Jianhua Liu, Shanmei Wang, Yushan Pan, Li Yuan, Gongzheng Hu

**Affiliations:** 1 College of Animal Husbandry and Veterinary Science, Henan Agricultural University, Zhengzhou, 450002 China; 2 Medical Microbiology Laboratory, Henan Provincial People’s Hospital, Zhengzhou, 450002 China

**Keywords:** *OqxAB*, Multidrug efflux pump, Antimicrobial susceptibility, Southern hybridization, Conjugation experiments

## Abstract

**Background:**

The plasmid-encoded multidrug efflux pump *oqxAB* confers bacterial resistance primarily to olaquindox, quinolones, and chloramphenicol. The aims of this study were to investigate the prevalence of *oqxAB* among *Escherichia coli* isolates from dogs, cats, and humans in Henan, China and the susceptibilities of *E. coli* isolates to common antibiotics.

**Methods:**

From 2012 to 2014, a total of 600 samples which included 400 rectal samples and 200 clinical human specimens were tested for the presence of *E. coli*. All isolates were screened for *oqxAB* genes by PCR and sequencing. The MICs of 11 antimicrobial agents were determined by the broth microdilution method. A total of 30 representative *oqxAB*-positive isolates were subjected to ERIC-PCR and MLST. Additionally, conjugation experiments and southern hybridizations were performed.

**Results:**

Of 270 isolates, 58.5% (62/106) of the isolates from dogs, 56.25% (36/64) of the isolates from cats, and 42.0% (42/100) of the isolates from humans were positive for the *oqxAB*. Olaquindox resistance was found for 85.7%-100% of *oqxAB*-positive isolates. Of *oqxAB*-positive isolates from dogs, cats, and humans, ciprofloxacin resistance was inspected for 85.8%, 59.1%, and 93.8%, respectively. Several *oqxAB*-positive isolates were demonstrated by ERIC-PCR and MLST, and have high similarity. Phylogenetic analysis showed that *oqxAB*-positive isolates could be divided into 7 major clusters. *OqxAB*-positive conjugants were obtained, southern hybridization verified that the *oqxAB* gene complex was primarily located on plasmids.

**Conclusion:**

In conclusion, *oqxAB*-positive isolates were widespread in animals and humans in Henan, China. Carriage of *oqxAB* on plasmids of *E. coli* isolates may facilitate the emergence of multidrug resistant and its transmission via horizontal transfer, and might pose a potential threat to public health.

**Electronic supplementary material:**

The online version of this article (10.1186/s13756-018-0310-8) contains supplementary material, which is available to authorized users.

## Background

Nowadays, the growing frequency of antibiotic resistances is a universal problem. Antimicrobial resistance occurs through various mechanisms, such as drug efflux pumps [1, 2]. A novel multidrug efflux pump of Gram-negative bacteria, *oqxAB*, is a member of the root-nodulation-cell-division (RND) family and was first identified as being encoded by a plasmid-mediated gene that conferred resistance to olaquindox [3]. A number of studies have reported on the occurrence of *oqxAB* genes primarily in *Enterobacteriaceae*, including *Escherichia coli, Enterobacter cloacae*, and *Klebsiella pneumoniae* isolated from pigs, chickens, humans, and the environment [4–9]. However, the highest positive rates of *oqxAB* were found in surveys of animals in China [6, 7], and the primary reason might be the widespread use of olaquindox as a production animal growth enhancer [10]. It has been convincingly demonstrated that the genes *oqxAB* can be horizontally transferred among food-borne pathogens and confer antimicrobial resistance for a variety of antimicrobials, such as quinolones, and chloramphenicol [11, 12]. Because these antimicrobials are a significant part of drug therapy to some human bacterial infections, resistance to these drugs could ultimately pose a significant threat to human health. Thus, investigating prevalence of *oqxAB* genes in pathogenic *E. coli* isolates in China is paramount for establishing guidelines for veterinary and human clinical medication use.

Because of the close relationships between humans and their pets, an investigation of multidrug efflux pumps, especially those encoded by the identified *oqxAB* genes, would be of particular significance for medical science. The aim of this study was to investigate the prevalence of the *oqxAB* genes among *E. coli* isolates from companion animals and humans in Henan Province, China.

## Methods

### Sampling and bacterial isolates

From March 2012 to July 2014, a total of 400 rectal swab samples were recovered from 400 diseased pets (200 dogs and 200 cats) with symptoms of fever, diarrhea and respiratory diseases at three animal hospitals in Henan Agricultural University, ZhengZhou. A total of 200 blood specimens were recovered from adult humans with symptoms of fever, bacteraemia and diarrhea at Henan Provincial People’s Hospital during October 2012 and August 2014. All samples were immediately transported to the laboratory under required preservation conditions (in a cooler with ice) within 6 h of collection, and processed within 2 h for samples to test the presence of *E. coli*. The samples were incubated in LB media (Beijing Land Bridge Technology Co., Ltd., Beijing, China) at 37 °C overnight for 16-20 h, and draw the line on MacConkey agar plateafter dipping the culture the next day. All presumptive *E. coli* colonies were identified using VITEK 2 compact automated identification system (BioMérieux, Marcy-I’Etoile, France).

### Determination of *oqxAB* and insertion sequences

A total of 270 strains were screened for *oqxA* and *oqxB* genes by PCR using specific primers as described previously [5]. The amplicons obtained were confirmed by sequencing. The whole coding region of the *oqxAB* gene complex in representative strains isolated from three origins were amplified using primer pairs (producing a fragment of 5140 bp), as reported previously [6]. Then, a PCR product was ligated to a pUC18-T vector (TaKaRa Biotechnology, Dalian, China) and bi-directionally sequenced.

Additionally, the association of insertion sequences *ISEcp1* and *IS26* with *oqxA* were also investigated in all *oqxAB*-positive isolates by PCR using the forward primers *ISEcp1*-F (5’-GGCCACGTGCATTTTTTATT-3′) and *IS26*-F (5’-AGCGGTAAATCGTGGAGTGA-3′) and the reverse primer *oqxA*-R (5’-TCAGGTGAATGTTTCCCCAG-3′) located in *oqxA*, respectively. The PCR products were sequenced and analyzed with BLAST program to confirm correct amplification.

### Antimicrobial susceptibility testing

The minimum inhibitory concentrations (MICs) of 11 antimicrobial agents (Additional file 1: Table S1) against the 270 *E. coli* strains, were determined by the broth microdilution method according to the recommendations of the Clinical and Laboratory Standards Institute (CLSI) [13]. *E. coli* ATCC 25922 was used as a reference strain for quality control in the MIC determinations. The MIC_50_ and MIC_90_ were determined which represent concentrations of the relevant antibiotics which inhibited growth of the bacteria by 50% or 90% respectively. The MIC breakpoints for most antimicrobial agents were in accordance with CLSI [13, 14]. The MIC breakpoints for olaquindox and mequindox were based on relevant references [3, 4]. But, if CLSI criteria were not available for some antibiotics, the results were interpreted according to criteria of the European Committee on Antimicrobial Susceptibility Testing (EUCAST) [15].

### Molecular typing

A total of 30 representative *oqxAB*-positive *E. coli* strains (olaquindox-resistant and MIC≥128 μg/mL) selected randomly from humans, dogs and cats were subjected to Enterobacterial Repetitive Intergenic Consensus PCR (ERIC-PCR) using the primers [16]: ERIC-1, 5’-ATGTAAGCTCCTGGGGATTCAC-3′ and ERIC-2, 5’-AAGTAAGTGACTGGGGTGAGCG-3′. Template DNA was extracted using the conventional boiling method, the sample was heated in a thermocycler at 100 °C for 10 min. Immediately, the sample was incubated at − 20 °C for 15 min, and was centrifuged at 12000×g for 15 min. The size of the amplified fragments ranged from 300 to >3000 bp. A similarity coefficient greater than 80% was used to define the same subtype.

Multilocus Sequence Typing (MLST) of these 30 representatives was done by PCR and sequencing. Seven housekeeping genes (*aspC*, *clpX*, *fadD*, *icdA*, *lysP*, *mdh*, and *uidA*) were amplified and sequenced. The corresponding types (STs) were matched using the electronic database on the *E. coli* MLST website (http://www.mlst.net/). A phylogenetic tree for the 7 housekeeping gene sequences was constructed using Phylip 3.69 software and their affinity relationships were described.

The phylogenetic groups of the 30 isolates mentioned above were determined by the multiplex PCR-based method, as previously described [17].

### Conjugation experiments

Conjugation experiments were done in LB broth or on filters with rifampicin-resistant *E. coli* C600 as the recipient [18]. Ten *oqxAB*-positive isolates were randomly selected among 30 *oqxAB*-positive strains and used as donor strains. Transconjugants were selected on LB agar or MacConkey plates containing olaquindox (64 μg/mL) and rifampicin (360 μg/mL). Antimicrobial susceptibility and detection of transferred *oqxAB* genes were performed for transconjugants.

### Southern hybridization testing

Southern hybridizations were used to test for the plasmid location of *oqxAB*, according to the procedures as described previously [19]. Plasmid DNA from *E. coli* was extracted using a Plasmid Midi Kit (QIAGEN, Valencia, CA), according to the manufacturer’s instructions. Plasmid DNA bands were then transferred and cross-linked onto a nylon membrane and were hybridised with digoxigenin (DIG) labeled *oqxAB* probes using the DIG high prime DNA labeling and detection starter kit (Roche, Mannheim, Germany).

### Nucleotide sequencing and submission of *oqxAB* sequences

The complete nucleotide sequences of the *oqxAB* genes of *E. coli* strains Q63, M50, and H050 were submitted to GenBank and were given the accession numbers JX294475, JX412478, and JX469117, respectively.

### Data and statistical analysis

The 270 isolated strains were categorized as sensitive (S), resistant (R) based on the MIC values and the CLSI interpretive criteria. For statistical analysis, we carried out chi-square test. A two-sided *p*-value ≤0.05 were considered to be statistically significant. SPSS 20.0 software (IBM, USA) was used for data analysis.

## Results

### Isolation and identification of *E. coli*

A total of 270 *E. coli* isolates were obtained, which included 106, 64, and 100 *E. coli* respectively collected from 200 rectal swab samples of dogs, 200 rectal swab samples of cats, and 200 clinical blood samples of humans.

### Prevalence of *oqxAB*

As shown in Table 1, 58.5% (62/106) of the isolates from dogs, 56.25% (36/64) of the isolates from cats, and 42.0% (42/100) of the isolates from humans were positive for the *oqxAB*. Two common insertion sequences, *ISEcp1* and *IS26*, were investigated in all *oqxAB*-positive isolates. The whole coding region of the *oqxAB* genes were amplified and sequenced. *ISEcp1*, truncated by *IS26*, was also observed in some *oqxAB*-positive strains (77/140, 55.0%). Interestingly, most of the *oqxAB* cassettes of the *oqxAB*-positive strains (111/140, 79.3%) were also flanked by *IS26* similar to composite transposon *Tn6010* of *K. pneumoniae*, which suggested that the dissemination of *oqxAB* among different *E. coli* strains might be mediated by the mobile element. This genetic organization (*ISEcp1-IS26*-*oqxAB*) is almost identical to corresponding sequences of initially identified plasmid pOLA52 carrying *oqxAB* genes. The *ISEcp1* and *IS26* upstream sequences identified here was the first to be associated with the *oqxAB* genes in *E. coli* from companion animals in Henan Province, China.

**Table 1:** Prevalence of *oqxAB* genes and susceptibility to 11 antimicrobial agents

Antibiotics^b^	Isolates from Dogs (*n* = 106)							Isolates from Cats (*n* = 64)							Isolates from Humans (*n* = 100)							Total *P*-value
	oqxAB-positive 58.5% (62//106)^a^			oqxAB-negative 41.5% (44//106)				oqxAB-positive 56.25% (36//64)^a^			oqxAB-negative 43.75% (28/64)				oqxAB-positive 42.0% (42//100)^a^			oqxAB-negative 58.0% (58/100)				
	MIC_50_	MIC_90_	R(%)	MIC_50_	MIC_90_	R(%)	*P*-value	MIC_50_	MIC_90_	R(%)	MIC_50_	MIC_90_	R(%)	*P*-value	MIC_50_	MIC_90_	R(%)	MIC_50_	MIC_90_	R(%)	*P*-value	
COL	0.25	1	0	0.25	1	0	–	0.125	0.25	0	0.03	0.25	0	–	0.25	8	15.6	0.5	4	11.1	0.505	0.674
TET	128	>256	95.2	32	64	72.7	0.054	64	256	100	16	64	80	0.091	32	64	100	16	64	72.2	0.004	6.63 × 10^−5^
OLA	64	128	85.7	16	64	18.2	4.77 × 10^−5^	64	256	100	2	64	20	0.008	64	128	90.6	4	32	11.1	2.69 × 10^−8^	5.79 × 10^−12^
MEQ	4	64	–	1	4	–	–	16	64	–	4	16	–	–	32	128	–	4	16	–	–	–
CEF	16	64	73.8	8	32	72.7	0.608	8	32	77.3	8	64	80	0.624	128	>512	96.9	128	>512	94.4	0.595	0.745
CIP	32	>512	85.8	<0.25	4	36.4	0.002	4	32	81.8	0.25	4	30	0.007	64	>512	93.8	8	256	66.7	0.019	1.57 × 10^− 6^
CRO	64	>512	73.8	8	32	63.6	0.375	4	32	59.1	8	64	70	0.427	64	>512	90.6	128	>512	94.4	0.544	0.704
DOX	64	>128	88.1	8	16	54.5	0.023	32	>128	81.8	4	16	70	0.376	16	128	93.8	16	64	88.9	0.456	0.039
GAT	8	64	57.5	0.5	4	18.2	0.021	2	8	59.1	<0.25	8	30	0.127	16	64	87.5	8	64	44.4	0.001	2.47 × 10^−4^
AMK	32	>512	61.9	16	512	81.8	0.191	0.25	8	13.6	2	16	30	0.264	256	512	84.4	256	>512	88.9	0.505	0.144
FFC	16	128	78.6	4	128	63.6	0.257	4	16	68.2	2	16	40	0.138	128	256	87.5	64	256	66.7	0.083	0.016

-, no statistical or not determined

^a^Rate of oqxAB-positive isolates; oqxAB-positive isolates/t total isolates

^b^*COL* colistin, *TET* tetracycline, *OLA* olaquindox, *MEQ* mequindox, *CEF* ceftiofur; *CRO* ceftriaxone, *DOX* doxycyclin, *CIP* ciprofloxacin, *GAT* gatifloxacin, *AMK* amikacin, *FFC* florfenicol

### Antimicrobial susceptibility testing

The MICs of 11 common antibiotics against the 270 *E. coli* isolates from dogs, cats, and humans are shown in Table 1. The MIC_50_ values of olaquindox for *oqxAB*-positive strains were 4 to 16-fold higher than those for *oqxAB*-negative strains (*p* < 0.01). The MIC_50_ (1-32 μg/mL) and MIC_90_ (4-128 μg/mL) values of mequindox were 2 to 8-fold lower than those for olaquindox against both *oqxAB*-positive and *oqxAB*-negative isolates. The MIC_50_ values of ciprofloxacin and florfenicol for *oqxAB*-positive isolates were all 2 to 16-fold higher than those for *oqxAB*-negative isolates from the three different origins. Importantly, the MIC_50_ values of ceftiofur and ceftriaxone were 4-128 μg/mL for both *oqxAB*-positive and *oqxAB*-negative isolates, which were much higher than the breakpoints (≤2 μg/mL) for susceptibility. By comparison, the MIC_50_ values of colistin were 0.125-0.5 μg/mL, which were much lower than the breakpoints (< 2 μg/mL) for susceptibility. Moreover, all isolates from companion animals were susceptible to colistin.

Among *oqxAB*-positive *E. coli* strains, resistance rates of ≥68.2% were found for six antibiotics (olaquindox, tetracycline, florfenicol, ceftiofur, doxycycline, and ciprofloxacin). The resistance rates of the *oqxAB*-positive isolates from dogs and humans to olaquindox were 85.7% and 90.6%. However, the resistance rates to tetracycline, olaquindox, ciprofloxacin, and gatifloxacin were significantly higher among *oqxAB*-positive isolates than among *oqxAB*-negative isolates (*p* < 0.01). Of a total of 270 *E. coli* strains, more than 59.1% strains were resistant at the same time to ceftiofur and ceftriaxone. However, there were no significant differences in the resistance rates between the *oqxAB*-positive and *oqxAB*-negative strains (*p* > 0.05).

### ERIC-PCR, MLST, and phylogenetic analysis

A total of 13 genotypes were identified by ERIC-PCR homology analysis from 30 *oqxAB*-positive *E. coli* isolates. Eight isolates (26.7%) primarily belonged to genotype III, followed by genotypes I (7 strains; 23.3%), VI (5 strains; 16.7%), and VIII (4 strains; 13.3%). The remaining each genotypes were found in 1-3 isolates.

**Fig. 1:**
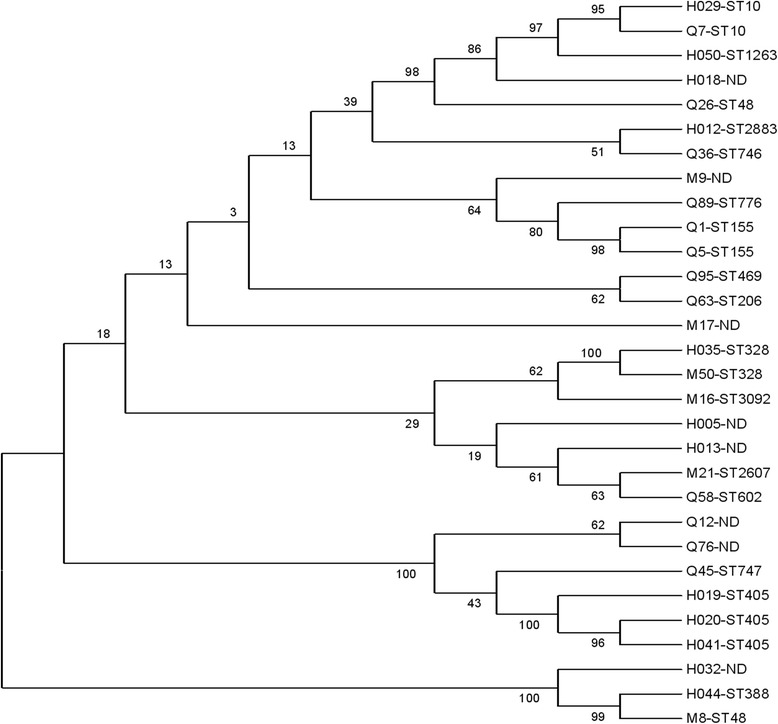
Dendrogram showing the genetic relatedness of 30 *E. coli* strains (oqxAB-positive). A phylogenetic tree for the seven housekeeping gene sequences was constructed using Phylip 3.69 software and their affinity relationships were described. Notes: Number “M” strains isolated from cats; Number “Q” strains isolated from dogs; Number “H” strains isolated from humans; ND, not determined

A total of 17 MLST types were identified among these 30 strains from different origins (Fig. 1). Nine novel sequence types (ST3081, ST3082, ST3092, ST3093, ST3094, ST3095, ST3097, ST3098 and ST3099) were detected in this study. The *E. coli* isolates from dogs, cats, and humans that exhibited identical ERIC-PCR patterns also showed coincident sequence types, for instance, M8 and Q26 (ST48), H029 and Q7 (ST10), Q1 and Q5 (ST155). As shown in Fig. 1, *oqxAB*-positive isolates could be divided into seven major clusters.

Phylogenetic analysis showed that 30 representative strains belonged to group A (23.3%), group B1 (30.0%), group B2 (26.7%), and group D (20.0%), respectively.

### Transfer of antimicrobial resistance and the *oqxAB* genes

Eight transconjugants were successfully obtained from 10 *oqxAB*-positive isolates by conjugation experiments. Two isolates from dogs did not yield transconjugants. As shown in Table 2, the MICs of mequindox and olaquindox for all transconjugants were similar to those observed for the donors, but were about 8 to 64-fold higher than those observed for the recipients. The *oqxAB* genes in these 8 *oqxAB*-positive isolates and mequindox and olaquindox resistance were transferred in the conjugation experiments.

**Table 2:** MICs for transconjugants and characterization of plasmids carrying *oqxAB*

Strains	Donors												Plasmid		Transconjugants							
	origin	MLST	ERIC-PCR	Phylogenetic group	MICs (μg/mL)								Amount	Size(kb)	MICs (μg/mL)							
					COL	OLA	MEQ	CEF	CIP	DOX	AMK	FFC			COL	OLA	MEQ	CEF	CIP	DOX	AMK	FFC
Q1	Dog	ST155	I type	A	0.5	512	32	<0.25	64	32	1	4	ND	ND	0.5	256	32	<0.25	4	2	1	4
M17	Cat	ST48	VI type	B1	0.5	256	64	32	8	32	256	64	1	54~ 108	0.5	256	32	32	1	16	4	8
Q63	Dog	ST206	II type	B2	0.25	256	16	8	1	8	64	128	2	54~ 108	0.5	256	16	<0.25	1	4	8	32
H050	Human	ST1263	VII type	A	0.125	256	64	256	64	16	512	128	2	54~ 108	0.25	512	64	32	8	32	64	16
H029	Human	ST10	V type	D	0.125	256	64	128	32	16	512	64	1	54~ 108	0.125	256	64	64	4	32	32	32
H035	Human	ST328	III type	B2	0.5	256	32	0.5	64	16	1	8	ND	ND	0.25	512	16	32	4	32	8	8
M50	Cat	ST328	III type	D	0.25	256	32	0.25	0.25	4	0.25	4	ND	ND	0.25	256	16	<0.25	<0.25	16	0.25	2
Q76	Dog	ND	IV type	B2	4	512	128	256	16	32	256	128	ND	ND	0.25	256	128	128	2	16	32	16
C600															0.25	4	4	0.06	0.03	0.25	0.5	1

*ND* not determined, *COL* colistin, *OLA* olaquindox, *MEQ* mequindox, *CEF* ceftiofur, *DOX* doxycycline, *CIP* ciprofloxacin, *AMK* amikacin, *FFC* florfenicol

C600, recipients in conjugation experiments

The MICs of florfenicol for the *oqxAB* transconjugants ranged from 2 to 32 μg/mL, which were about 2 to 32-fold higher than those obtained for the recipients. The MICs of colistin, ceftiofur, and doxycycline for transconjugants (except of J-M50) and donors were all similar and showed distinct increases as compared to recipients.

### Localization of the *oqxAB* genes

**Fig. 2:**
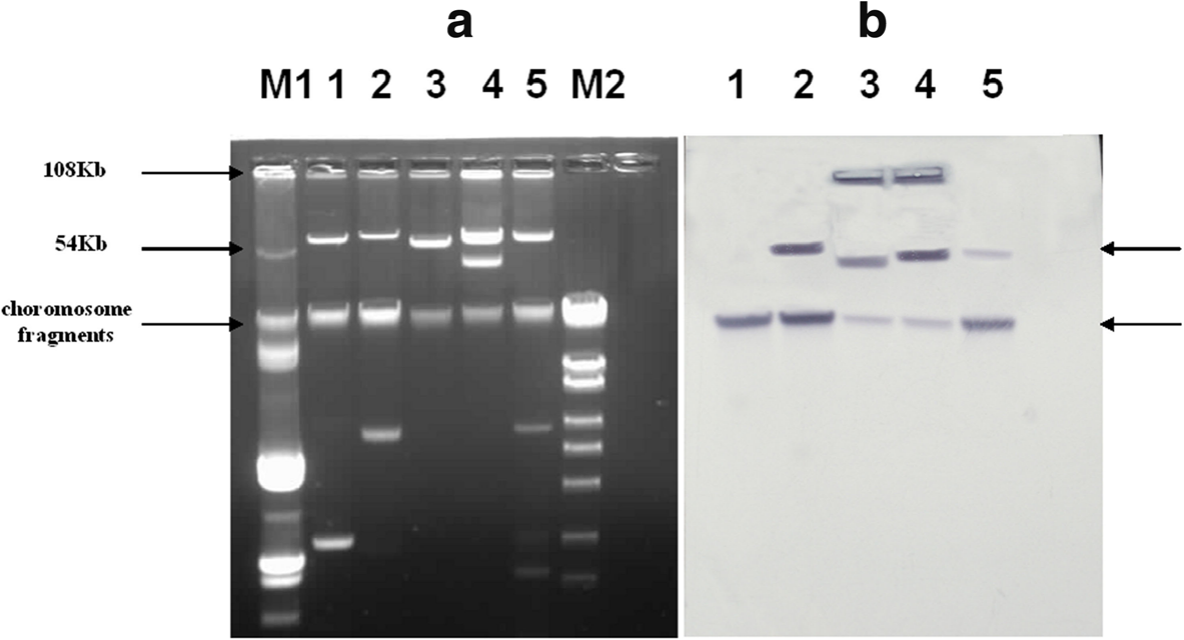
Agarose gel electrophoresis (**a**) and Southern hybridization (**b**) results for plasmid DNA preparations. Notes: Lanes M1, plasmid Marker V517; 1 to 5, *E. coli* Q1, M17, Q63, H050, and H029, respectively; M2, Marker 5000. Upper arrow in (**b**) indicates plasmid DNA; lower arrow in (**b**) indicates the location of chromosomal DNA and/or sheared plasmid DNA

Southern hybridization results (Fig. 2b) indicated that *oqxAB* genes were primarily found in plasmids with sizes of approximately 54 kb, except one strain (Q1 isolated from dog). It was remarkable that the isolates Q63 (isolated from dog) and H050 (isolated from human) yielded two distinct signals located on two plasmids of approximately 54 kb and another unknown size (at least 108 kb), respectively.

## Discussion

This is the first study to investigate the prevalence and dissemination of *oqxAB* in *E. coli* strains isolated from companion animals and from humans in Henan Province, China. It was surprising to find that there was a high prevalence of *oqxAB* in clinical isolates (51.85%), which was higher than those reported previously in Korea, Denmark, Sweden, Taiwan, and China from food animals and humans (0.06-46.3%) [3–7, 9, 11, 20, 21]. However, the prevalence of *oqxAB*-positive isolates in this study was lower than previously reported in Iran from urinary tract infections in humans (69.1%) [8]. The major reason may be that olaquindox has been extensively used as a growth promoter for food animals in China.

In agreement with previous studies [5, 11], 79.3% of the *oqxAB* cassettes of the *oqxAB*-positive isolates in this study were also flanked by *IS26* element similar to *Tn6010* of *K. pneumoniae*, suggesting that these mobile genetic elements are responsible for the dissemination of *oqxAB* genes [5, 22]. Interestingly, *ISEcp1* truncated by *IS26* was also observed in 55.0% of *oqxAB*-positive strains. This genetic organization (*ISEcp1-IS26*-*oqxAB*) is almost identical to corresponding sequences of initially identified plasmid pOLA52 carrying *oqxAB* genes [11]. This suggested that *IS26* was inserted in these strains at 3′ end of *ISEcp1* as a result of recombinatorial events.

However, this could not account for the significantly high prevalence of *oqxAB* in clinical isolates from humans, as olaquindox has not been overused for human clinical treatments in China. Probably, *oqxAB* genes were mostly located on transferable plasmids and could diffuse quickly between human and pet isolates through the food chain [5, 6, 11].

Mequindox, a new synthetic quinoxaline 1,4-dioxide (QdNO) derivative, was developed and widely used in China during the 1990s [10]. It has the same effects as olaquindox as a common animal feed additive to increase the economic benefits of breeding industry. In Table 1, the MIC_50_ values of mequindox were much lower than those for olaquindox, which differed from a previous report from China [6], and may have been due to its rare use in pets and its limited use in humans.

Excessive antimicrobial use in animals is considered to be the most important contributor to the selection of resistant bacteria [23]. Recently, the use of colistin should be limited in food and companion animals due to reduce the occurrence of *MCR*-1-harboring strains [24, 25]. Moreover, the use of olaquindox and mequindox also should be limited in food animals due to the high prevalence of the *oqxAB* gene that we found in China [16]. However, this study demonstrates that *oqxAB*-positive isolates were not all resistant to olaquindox, which is inconsistent with previous reports [4–7]. This is most probably related to the regulation of *oqxAB* genes expression. Additionally, it is possible that the *oqxAB* genes are either poorly expressed or not expressed in these isolates.

The diverse ERIC-PCR patterns among certain *oqxAB*-positive strains from different origins implied that the horizontal transmission of *oqxAB* was a possible determinant rather than the direct clonal dissemination between pets and humans, which is consistent with previous reports [26]. A total of 17 MLST types were identified among these 30 *oqxAB*-positive *E. coli* isolates from different origins, the same STs of *E. coli* strains from different origins suggested clonal dissemination of *oqxAB*-positive strains. Previously, a high prevalence of *oqxAB* in *E. coli* isolates associated with predominantly ST238 was reported [27]. However, recent reports showed that the *oqxAB*-positive *E. coli* isolates in all belonged to ST533 [28]. In view of this, further studies are needed to investigate the possible transmission of *oqxAB* genes by either the food chain or by co-infection.

Conjugation experiments and Southern blotting indicated that *oqxAB* genes were primarily located on plasmids (except of one strain Q1), which is consistent with previous reports [3–7, 11, 29]. However, in this study it was remarkable that there were two hybridization signals found on two plasmids (Fig. 2b) for one isolate from dog and one from human, which indicated they could simultaneously exist on plasmids of different sizes. Meanwhile, *oqxAB* conferred not only resistance to quinoxalines and chloramphenicol, but also reduced susceptibility to other antimicrobials such as florfenicol and fluoroquinolones. Carriage of *oqxAB* on plasmids of *E. coli* isolates may facilitate the emergence of multi-antibiotc resistance and its transmission via horizontal transfer, might pose a potential threat to public health and need for vigilant monitoring these isolates at the human-animal interface.

## Conclusion

In conclusion, *oqxAB*-positive isolates were widespread in pets and humans in Henan Province, China, and the prevalence of *oqxAB* genes were significantly higher than what was previously found in other countries or areas. Carriage of *oqxAB* on plasmids of *E. coli* isolates may facilitate the emergence of multidrug resistant and its transmission via horizontal transfer. The same STs of *E. coli* strains from different origins suggested clonal dissemination of *oqxAB*-positive strains. Probably, *oqxAB* genes were mostly located on transferable plasmids and could diffuse quickly between human and pet isolates through the food chain, this needs to further strengthen supervision and resistance detection. More attention should be paid to the transmission of *oqxAB* genes in the future.

## Additional file

Additional file 1: Table S1. A list of the eleven tested antimicrobials, their classes, their concentrations, and their breakpoints used for susceptibility testing of *E. coli*. (DOC 58 kb)

## Abbreviations

CLSI: Clinical and laboratory standards institute
EUCAST: European committee on antimicrobial susceptibility testing
MICs: Minimum inhibitory concentrations
MLST: Multilocus sequence typing
