# Supplementary material for: Prevalence and molecular characterization of oqxAB in clinical Escherichia coli isolates from companion animals and humans in Henan Province, China
Source: Antimicrob Resist Infect Control. 2018 Feb 2;7:18. doi: 10.1186/s13756-018-0310-8 (PMC5797404; doi:10.1186/s13756-018-0310-8)
Supplement: Additional file 1: Table S1. — A list of the eleven tested antimicrobials, their classes, their concentrations, and their breakpoints used for susceptibility testing of E. coli. (DOC 58 kb) [file 13756_2018_310_MOESM1_ESM.doc]

TABLE S1 A list of the eleven tested antimicrobials, their classes, their concentrations, and their breakpoints used for susceptibility testing of *E. coli*

| Antimicrobial categories | Antibiotics | Abbreviation | Resistance breakpoints (μg/mL) ^a^ | Senstivity breakpoints (μg/mL) ^a^ | Content (%) | Concentration range (μg/mL) |
| --- | --- | --- | --- | --- | --- | --- |
| Cephalosporins | ceftriaxone | CRO | ≥8 | ≤2 | 90% | 0.25-512 |
|  | ceftiofur | CEF | ≥8 | ≤2 | 98% | 0.25-512 |
| Fluoroquinolones | ciprofloxacin | CIP | ≥4 | ≤1 | 88.4% | 0.25-512 |
|  | gatifloxacin | GAT | ≥8 | ≤2 | 85% | 0.0625-128 |
| Tetracyclines | tetracycline | TET | ≥16 | ≤4 | 87.8% | 0.125-256 |
|  | doxycyclin | DOX | ≥16 | ≤4 | 80% | 0.0625-128 |
| Quinoxalines | olaquindox^b^ | OLA | ≥64 | ≤16 | 80% | 0.25-512 |
|  | mequindox^b^ | MEQ | ≥64 | ≤16 | 80% | 0.25-512 |
| independent class of antibiotics | colistin^c^ | COL | ≥2 | <2 | 90% | 0.015-32 |
| Aminoglycosides | amikacin | AMK | ≥64 | ≤16 | 65% | 0.25-512 |
| Amphenicols | florfenicol | FFC | ≥32 | ≤2 | 98% | 0.25-512 |

^a^ MIC (µg/mL) results were calculated according to CLSI (2013, 2016) breakpoint criteria [1, 2]; ^b^ the breakpoints for olaquindox and mequindox were based on Reference values [3, 4]; ^c^ the breakpoints for colistin was interpreted according to criteria of the European Committee on Antimicrobial Susceptibility Testing (EUCAST) [5].

**References**

1. **Clinical and Laboratory Standards Institute.** Performance Standards for Antimicrobial Disk and

Dilution Suseptibility Tests for Bacteria Isolated From Animals; Approved Standard-Fouth Edition. CLSI

document VET01-A4. CLSI, 2013; Wayne, PA, USA.

2. **Clinical and Laboratory Standards Institute.** Performance Standards for Antimicrobial Susceptibility

Testing; Twenty-Six Informational Supplement M100-S26, CLSI, 2016; Wayne, PA, USA.

3. **Hansen LH, Johannesen E, Burmølle M, Sørensen AH, Sørensen SJ.** Plasmid-encoded

multidrug efflux pump conferring resistance to olaquindox in *Escherichia coli*. *Antimicrob Agents*

*Chemother* 2004;48:3332-3337.

4. **Hansen LH, Sørensen SJ, Jorgensen HS, Jensen LB.** The prevalence of the *oqxAB* multidrug

efflux pump amongst olaquindox-resistant *Escherichia coli* in pigs. *Microb Drug Resist* 2005;11:378-

382.

5. **The European Committee on Antimicrobial Susceptibility Testing (EUCAST criteria).** Breakpoint

tables for interpretation of MICs and zone diameters. Version 7.0, 2017;[http://www.eucast.org](http://www.eucast.org/).
